# Development and certification of a reference material for zearalenone in maize germ oil

**DOI:** 10.1007/s00216-021-03532-z

**Published:** 2021-07-21

**Authors:** Juliane Riedel, Sebastian Recknagel, Diana Sassenroth, Tatjana Mauch, Sabine Buttler, Thomas Sommerfeld, Sibylle Penk, Matthias Koch

**Affiliations:** Bundesanstalt für Materialforschung und -prüfung (BAM), Richard-Willstätter-Straße 11, 12489 Berlin, Germany

**Keywords:** Fusarium mycotoxin, Vegetable edible oil, Food analysis, European Reference Material, Quality assurance

## Abstract

Zearalenone (ZEN), an estrogenic mycotoxin produced by several species of *Fusarium* fungi, is a common contaminant of cereal-based food worldwide. Due to frequent occurrences associated with high levels of ZEN, maize oil is a particular source of exposure. Although a European maximum level for ZEN in maize oil exists according to Commission Regulation (EC) No. 1126/2007 along with a newly developed international standard method for analysis, certified reference materials (CRM) are still not available. To overcome this lack, the first CRM for the determination of ZEN in contaminated maize germ oil (ERM®-BC715) was developed in the frame of a European Reference Materials (ERM®) project according to the requirements of ISO Guide 35. The whole process of CRM development including preparation, homogeneity and stability studies, and value assignment is presented. The assignment of the certified mass fraction was based upon an in-house study using high-performance liquid chromatography isotope dilution tandem mass spectrometry. Simultaneously, to support the in-house certification study, an interlaboratory comparison study was conducted with 13 expert laboratories using different analytical methods. The certified mass fraction and expanded uncertainty (*k* = 2) of ERM®-BC715 (362 ± 22) μg kg^−1^ ZEN are traceable to the SI. This reference material is intended for analytical quality control and contributes to the improvement of consumer protection and food safety.

**Graphical abstract:**

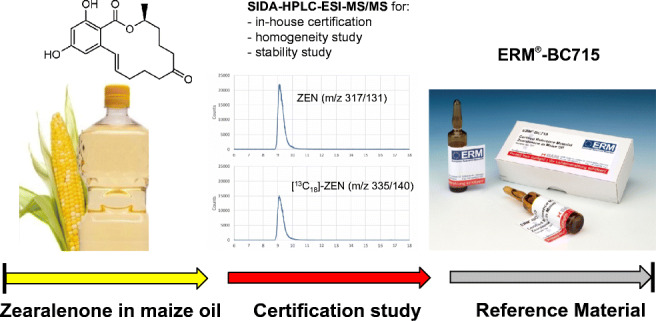

**Supplementary Information:**

The online version contains supplementary material available at 10.1007/s00216-021-03532-z.

## Introduction

Quality, safety and authenticity are key factors for placing confidence in food. Chemical food contaminants, which originate from different sources, lead to acute poisoning or have a long-term negative impact on the health of consumers. One of the most serious impacts to food safety issues arises from contaminated cereal crops, e.g. by heavy metals, pesticides and mycotoxins. The latter belong to the most abundant food contaminants worldwide. Estimates of food crops affected by mycotoxins worldwide vary widely, ranging from about 25% for levels above regulatory limits up to 60–80% for contamination levels above detection limits [1]. Mycotoxins lead in terms of annual notifications of the Rapid Alert System for Food and Feed (RASFF) and are responsible for most of the European border rejections [2, 3]. Due to serious toxic effects caused by mycotoxins, the surveillance, determination and reduction of these compounds in food and feed are subject to the work of legislative bodies, industry and chemical laboratories. In 2006, maximum levels were set for several mycotoxins in different food commodities [4].

**Fig. 1:**
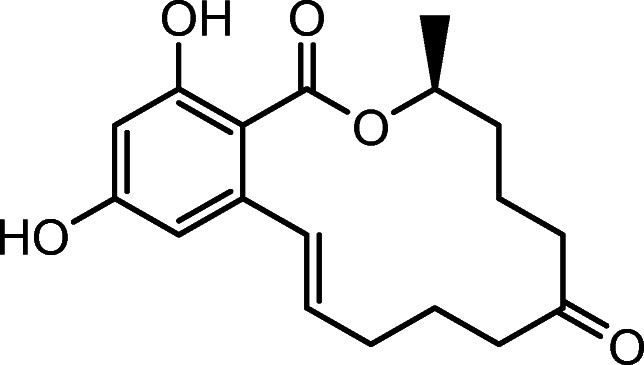
Zearalenone (ZEN)

Mycotoxins are secondary fungal metabolites. Zearalenone (ZEN), a macrocyclic lactone (Fig. 1), belongs to the most relevant mycotoxins contaminating cereal crops worldwide. Grains like wheat, barley, oats, sorghum and particularly maize are frequently contaminated by ZEN. It is biosynthesized by several *Fusarium* (*F*.) species including *F. graminearum*, *F. culmorum*, *F. cerealis* and *F. equiseti*. In vitro and in vivo studies demonstrate ZEN to be estrogenic, hepatotoxic, immunotoxic and carcinogenic [5, 6]. Previous findings [7, 8] suggest that vegetable edible oils, in particular maize germ oil, significantly contribute to the ZEN exposure, which was confirmed by the European Food Safety Authority (EFSA) [9].

In order to strengthen food safety and consumer protection, the EU amended the maximum level of ZEN in refined maize germ oil to 400 μg kg^−1^ [10] and issued a mandate to the European standardization body CEN for the development of analytical methods related to food mycotoxins with increasing relevance including ZEN [11]. The developed standard method for quantification of ZEN in vegetable edible oils is based on liquid/liquid (L/L) extraction followed by HPLC with fluorescence detection or, alternatively, mass spectrometric detection [12]. However, although a maximum level and a standard procedure are in force, there is no certified reference material (CRM) for the determination of ZEN in edible oils available to date.

Reference materials (RM) and especially CRM are useful tools to verify the accuracy of analytical measurements. Official food control laboratories are obliged to use validated methods whenever possible. Thus, analytical methods must be subject to validation to verify the performance of a method including accuracy, repeatability and reproducibility. But also non-official control laboratories, food manufacturers and researchers are interested in RM/CRM to provide accurate results. Since maize oils are the predominant source for ZEN among vegetable edible oils accompanied by a European regulatory value for refined maize oils, such a CRM has the most practical relevance

Thus, the aim of the presented project was to develop a CRM for ZEN in refined maize germ oil (ERM®-BC715) produced within the ERM® initiative (European Reference Materials). ERM®-BC715 is intended to be used for performance control and validation of analytical methods for the determination of ZEN in refined maize oils and similar vegetable edible oils. Thirteen laboratories were selected based on their documented experience and invited to participate in an interlaboratory comparison study (ILC) to support BAM’s in-house certification of the candidate material. Certification was carried out based on ISO 17034 [13] and the relevant ISO-Guides [14, 15].

Besides the EU limit for ZEN, there is also a maximum level for lead (Pb) in oils and fats (0.1 mg kg^−1^) [4]. Thus, a secondary matrix characterization of the candidate material was subjected to Pb and cadmium (Cd), which often co-occurs with Pb.

## Material and methods

### Material preparation

Based on the EU maximum level of 400 μg kg^−1^, the aim was to produce a refined maize germ oil CRM in a targeted range of 300–400 μg kg^−1^. Criteria to procure a suitable candidate material were (i) oil from a commercial source intended for human consumption and (ii) oil revealing a natural ZEN contamination. A survey of refined maize germ oils from German retail markets was conducted. Based on previous experiences, moderate up to high levels of ZEN could be expected. The procured samples were separately tested, and selected samples with suitable ZEN contents were combined (5.2 L) and thoroughly mixed. The initial analysis of the oil mixture revealed a ZEN content of about 350 μg kg^−1^.

To prevent a possible isomerization of the naturally occurring *trans*-ZEN to the photochemically induced *cis*-ZEN, amber glass ampoules (10 mL) were used to fill the CRM. Before bottling, the maize oil mixture was purged with argon for 5 h to remove residual oxygen and the empty ampoules were also flushed with argon to exclude oxygen. A total of 569 units were produced by filling a volume of 9 mL of oil. Ampoules were sealed immediately and stored at −20 °C.

### Analytical methods

All analyses within the CRM project (homogeneity, stability, certification) were carried out at BAM according to an accredited in-house method [16]. This method is based on high-performance liquid chromatography (HPLC) hyphenated to electrospray tandem mass spectrometry (HPLC-ESI-MS/MS) using a stable isotope dilution analysis (SIDA) approach.

#### ZEN analysis

The isotopically labelled internal standard [^13^C_18_]-ZEN solution in acetonitrile (Biopure™, Tulln, Austria) was weighed into a 15-mL centrifuge tube (Falcon™) followed by solvent evaporation in a gentle stream of nitrogen at 50 °C. Afterwards, 0.5 mL of oil sample was weighed into the Falcon™ tube and 0.5 mL n-hexane was added. After adding 5 mL of the extraction solution (methanol/water, 9/1, v/v), the tube was sealed and placed on a horizontal shaker for extraction (30 min at 400 min^−1^). The sample was centrifuged at room temperature at 2400 min^−1^ (1378 *g*) for 10 min. An aliquot of 1 mL from the upper, methanolic layer containing ZEN was transferred into a HPLC vial and evaporated to dryness in a gentle stream of nitrogen at 50 °C. The residue was dissolved in 0.4 mL of HPLC eluent (acetonitrile/water, 38/62, v/v) and analysed by HPLC-ESI-MS/MS.

The chromatographic separation was performed with an Agilent 1200 HPLC system (Agilent Technologies, Inc., Santa Clara, CA, USA) using a Gemini® NX C18 column (Phenomenex, Torrance, USA) with 150 × 2 mm (3-μm particle size) coupled to a Gemini® C18 guard column (2.0 × 4.0 mm). An isocratic HPLC eluent of (water + 0.1% formic acid) as mobile phase A and (acetonitrile + 0.1% formic acid) as mobile phase B was applied holding 62% A for 15 min. A sample volume of 10 μL was injected; the column oven was set to 50 °C. Mass spectrometric measurements were performed by means of a SCIEX Triple Quad API 4000 QTRAP® (AB Sciex LLC, Framingham, MA, USA) operating in electrospray ionization (ESI) multiple reaction monitoring (MRM) mode. The monitored mass transitions given in Table 1 were used for ZEN quantification of the candidate material.

**Table 1:** Mass transitions of native and isotopically labelled zearalenone (ZEN) for the HPLC-MS/MS analysis using ESI negative (−) MRM mode

Compound	MRM transition (m/z)	Dwell time (ms)	DP (V)	CE (eV)	CXP (V)
ZEN	317.1 [M-H]- → 131.1^a^	50	−80	−42	−8
ZEN	317.1 [M-H]- → 175.0^b^	50	−80	−40	−18
[^13^C_18_]-ZEN	335.2 [M-H]- → 140.2^a^	50	−80	−42	−7

*DP*, declustering potential; *CE*, collision energy; *CXP*, collision cell exit potential

^a^Quantifier transition

^b^Qualifier transition

Calibration was performed in a range between 80 and 500 μg kg^−1^ with six calibration points (regression coefficient *R*^2^ = 0.9984) using a certified ZEN standard (Biopure™, Tulln, Austria) as calibrant and [^13^C_18_]-ZEN as internal standard (IS). The measured area ratio of ZEN/IS was used for quantification. All calibration solutions were freshly prepared by weighing.

**Fig. 2:**
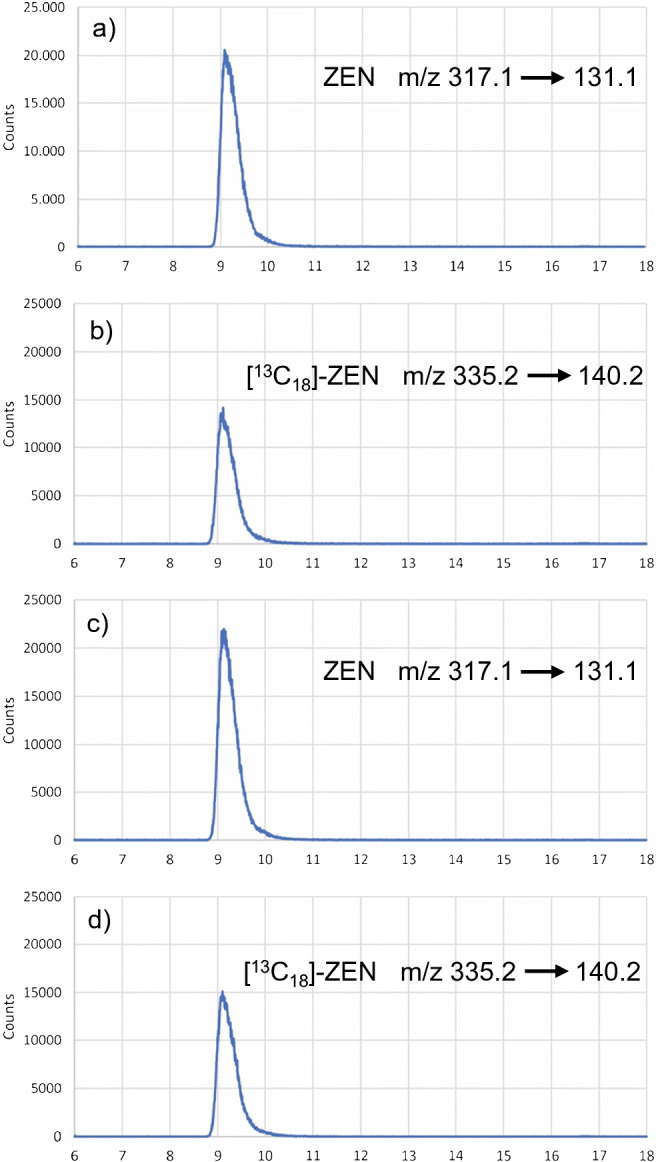
HPLC-ESI(-)-MS/MS chromatograms with mass transitions of ZEN and [^13^C_18_]-ZEN used for quantification; **a**, **b** calibration standard; **c**, **d** ERM®-BC715

The HPLC-MS/MS chromatograms of a calibration solution and an ERM®-BC715 sample are displayed in Fig. 2 showing the mass transitions for native ZEN and isotopic labelled ZEN as internal standard.

#### Lead and cadmium analysis

Seven subsamples of ERM®-BC715 (sample intake 0.26 g each) were dissolved in a fully automatic microwave digestion system (ULTRACLAVE, MLS Mikrowellen-Labor-Systeme GmbH, Leutkirch, Germany) using 4 mL of HNO_3_ (65%, cleaned by subboiling distillation, Merck, Darmstadt, Germany). The dissolution program comprised heating up to 255 C and 160 bar for 45 min. Trace element determination in the clear sample solutions was carried out with inductively coupled plasma mass spectrometry (ICP-MS) using a quadrupole ICP-mass spectrometer 7800 (Agilent Technologies, Inc., Santa Clara, CA, USA) equipped with a micro flow nebulizer and He as reagent gas in the reaction cell (plasma power: 1500 W). Calibration was performed using seven acidified standard solutions prepared from 1000 mg L^−1^ monoelement ICP standards (Merck Certipur, Merck, Darmstadt, Germany). All measurements were carried out using the ^111^Cd and ^208^Pb isotopes with ^72^Ge and ^165^Ho as internal standards.

## Homogeneity study

The homogeneity study assesses the distribution of the analyte (ZEN) in all the units bottled. That means that uncertainty contribution resulting from possible heterogeneity (between-unit inhomogeneity) has to be quantified. An estimate for the between-bottle inhomogeneity of the candidate CRM was assessed by determining ZEN in 20 units equidistantly selected from the whole set of the 569 units in the order of bottling. The selected units were processed five times each according to the analytical method described here. All extracts (20 × 5 = 100) were analysed by HPLC-MS/MS in a randomized manner under repeatability conditions in one run and quantified against one calibration.

The estimate of analyte-specific inhomogeneity contribution *u*_bb_ to be included in the overall uncertainty budget of ERM®-BC715 was calculated according to ISO Guide 35 [15] using Eq. 1:

1 $$ {u}_{\mathrm{bb}}=\sqrt{\frac{{\mathrm{MS}}_{\mathrm{between}}-{\mathrm{MS}}_{\mathrm{within}}}{n}} $$

where

*MS*_between_: mean of squared deviation between units (from one-factorial ANOVA)
*MS*_within_: mean of squared deviation within units (from one-factorial ANOVA)
*n*: number of replicate determinations per unit

### Stability study

An initial isochronous stability study [17] was conducted to quantify the mycotoxin degradation by subjecting candidate material units to accelerated ageing at temperatures between +4 and + 60 °C over periods of 0.25 to 12 months. After the respective periods of time, individual units were stored at −20 °C, a storage temperature that has been shown in previous RM projects to prevent further degradation of food mycotoxins. In addition, two ampoules each were stored at −20 °C and − 80 °C throughout the whole study period, where degradation of ZEN can be reasonably excluded. By this approach, the suitability of the commonly approved storage temperature of −20 °C should be confirmed. At the end of the stability study, all units were analysed simultaneously for ZEN under repeatability conditions.

Data processing and result assessment strictly followed the procedures as described by Bremser et al. [18] assuming an *Arrhenius* model for the dependence of the effective reaction rate *k*_eff_(*T*) on temperature. This kinetic approach was also used to estimate the shelf life (expiry date) of ERM®-BC715 as successfully done for other *Fusarium* mycotoxin CRMs [19, 20]. However, further investigations on stability of ERM®-BC715 are planned as part of the post-certification monitoring.

#### Characterization and value assignment

The assignment of the certified ZEN mass fraction of ERM®-BC715 was based upon an in-house study at BAM using a primary ratio method of SIDA-HPLC-ESI-MS/MS using [^13^C_18_]-ZEN as internal standard (see above). For in-house certification, 20 units of the candidate reference material were analysed five times each, resulting in a total of 100 independent analyses. It was accepted within the ERM® consortium (BAM, JRC, LGC) to use the data of the homogeneity study for assignment of the certified value.

In order to support the in-house certification data, an ILC was conducted by involving thirteen laboratories selected based on their approved expertise in the field of mycotoxin analysis. Each laboratory received two ampoules of ERM®-BC715 asked to provide six values, three independent replicates from each ampoule. The participants of the ILC applied methods of their own choice uniformly using a certified ZEN calibration standard (Biopure™, Tulln, Austria) provided by BAM.

The reported sample intake of the ILC participants was between 1 and 7.7 g (Table 2). The most commonly applied extraction method was L/L partition by shaking using acetonitrile/water or methanol/water mixtures (after dilution with n-hexane). Some laboratories used alkaline extraction mixtures to improve the solubility of nonpolar ZEN by deprotonation of the phenolic groups. If a clean-up step was applied (5 out of 13 labs), immunoaffinity column (IAC) was the method of choice to purify the extracts (4 out of 5 labs). Instrumental analysis was uniformly done by HPLC using either fluorescence detection (9 labs) or MS/MS detection (4 labs).

**Table 2:** Extraction, clean-up and determination methods used in the ILC for ERM®-BC715

Lab code	Sample (g)	Extraction method	Extraction solvent	Clean-up	Instrumental method	Internal standard
A	2.5	Shaking	ACN/water	No	HPLC-MS/MS	[^13^C_18_]-ZEN
B	1	Shaking	ACN/water	No	HPLC-MS/MS	No
C	1.5	Shaking	MeOH/water	No	HPLC-FLD	No
D	1–2	Shaking	MeOH/water	No	HPLC-FLD	No
E	1	Shaking	MeOH/water	IAC	HPLC-FLD	No
F	2	Shaking	MeOH/water	No	HPLC-FLD	No
G	2	Shaking	MeOH/water	IAC	HPLC-FLD	No
H	1.5	Shaking	ACN/water	IAC	HPLC-FLD	No
I	7.7	Shaking	ACN/water	IAC	HPLC-FLD	No
J	2	Shaking	MeOH/water	No	HPLC-FLD	No
K	1	GPC	CH/EtOAc	No	HPLC-MS/MS	[^13^C_18_]-ZEN
L	2.5	Shaking	ACN/water	SPE	HPLC-MS/MS	[^13^C_18_]-ZEN
M	2	Shaking	MeOH/water	No	HPLC-FLD	No

## Results and discussion

The certification campaign of ERM®-BC715 implied homogeneity evaluation, stability testing, in-house characterization for assignment of the certified value and ILC, and calculation of the total uncertainty budget also enabling a statement of traceability.

### Assessment of homogeneity

**Fig. 3:**
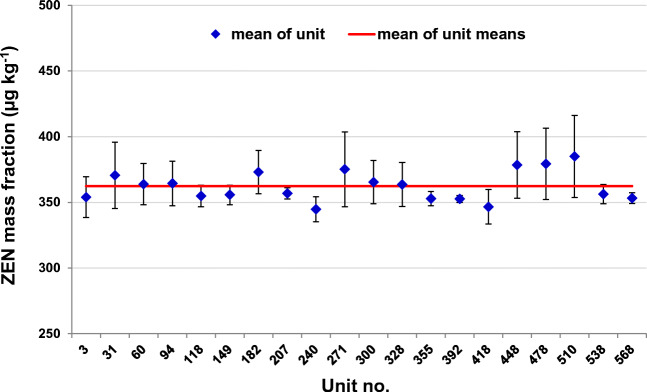
Homogeneity study of ERM®-BC715: mean values of ZEN for 20 selected units with their corresponding standard deviations (*n* = 5) represented by error bars. The mean of all unit means (red line) is 362.3 μg kg^−1^

Based upon the results of preliminary studies with liquid reference materials and thorough batch homogenization, a satisfactory level of homogeneity for ERM®-BC715 was expected. Furthermore, it could be assumed that ZEN as nonpolar substance (*K*_ow_ = −3.66) is dissolved in the nonpolar oil matrix with a satisfactory level of homogeneity. The results of the homogeneity study indicate that there is no trend regarding the ampoule filling order (Fig. 3).

The results were evaluated using one-factorial ANOVA (Table 3). Because the test statistic was slightly higher than the critical value, the candidate material was considered to be slightly inhomogeneous, but to an extent that can be covered by the corresponding uncertainty. According to ISO Guide 35, an inhomogeneity contribution to the total uncertainty has to be considered if MS_between_ is larger than MS_within_. Based on the ANOVA results and applying Eq. 1, the contribution *u*_bb_ to the overall uncertainty of ERM®-BC715 was calculated being *u*_bb_ = 8.463 μg kg^−1^.

**Table 3:** Analysis of variance (ANOVA) and estimate for uncertainty contribution for ZEN in ERM®-BC715 according to ISO Guide 35

MS_between_ (μg^2^ kg^−2^)	MS_within_ (μg^2^ kg^−2^)	*F*_obs_	*F*_crit_ (*f*_1_, *f*_2_, 5%)	*u*_bb_ (μg kg^−1^)
653.371349	295.264866	2.213	1.718	8.463

The obtained results are valid for oil sample intake of 0.5 mL (0.45 g). Therefore, test portions less than this minimum sample intake should not be used in order to avoid possible inhomogeneities with smaller sample sizes.

### Assessment of stability

From experience with ZEN in a wheat reference material [19], a temperature-driven deterioration of the mycotoxin content was to be expected also for maize germ oil. To quantify this effect, selected units were exposed to elevated storage temperatures taken after predefined sampling intervals. From semi-logarithmic plots of measured ZEN values over time, effective reaction rates *k*_eff_ were determined for each storage temperature. The dependence of ln(−*k*_eff_) on the inverse temperature (1/*T*) by assuming an *Arrhenius* model is displayed in Fig. S1 (Supplementary Information, ESM).

Temperature dependence can merely be approximated by a straight line. The calculated activation energy Δ*E* for ZEN (53.2 kJ mol^−1^) is in an acceptable agreement with activation energies determined for a large variety of organic compounds and is also similar to Δ*E* (ZEN) resulting from the stability study of ERM®-BC600 (Δ*E* = 58.0 kJ mol^−1^).

Using these data and the assumed *Arrhenius* model, it is possible to estimate when mycotoxin levels are expected to fall below the certified lower expanded uncertainty level as a result of degradation. In the sense of a worst-case estimation, these calculations are carried out for the reaction rates at the upper confidence limit of the regression line. Shelf life estimations for ZEN in ERM®-BC715 are stated in Table 4 for different storage temperatures.

**Table 4:** Estimated time in months up to which the certified ZEN value of ERM®-BC715 remains within the expanded uncertainty *U* at various storage temperatures

Temperature (°C)	Expiry (months)
−20	799
4	114
23	27
40	8
60	2

A storage temperature of −20 °C and even +4 °C is sufficient for a desirable minimum shelf life of 5 years. For this reason, an uncertainty contribution due to long-term (in)stability was not considered. Exposure to room temperature or higher than room temperature may reduce the shelf life of ERM®-BC715. Therefore, a common user expiration date of 2 years after delivery from storage is established, provided the sample is stored at +4 °C or below at the user’s premises. Our results are consistent with the outcome of the 2003 stability study for the ‘ZEN in maize’ CRM BCR-717. No significant degradation was observed for long-term stability, leading to a recommended storage temperature of +4 °C [21, 22]. Since BCR-717 (new code ERM®-BC717) is still available to date, high stability of ZEN in food matrices such as maize and maize oils can be expected. The first estimation of stability will continuously be updated by further measurements of units stored at −20 °C and + 4 °C throughout the period of availability of the material (post-certification monitoring).

### Characterization and value assignment

#### In-house study

It was agreed among the ERM® partners that the comprehensive homogeneity data derived using a primary ratio SIDA-HPLC-MS/MS method would be the basis for assigning the certified value. The ZEN mass fraction to be certified for ERM®-BC715 was determined to be 362.3 μg kg^−1^ based on the unweighted mean value of 20 ampoule mean values, with each unit analysed five times.

#### ILC

To better assess the results of the participants, each laboratory received two QC-solutions of ZEN in acetonitrile for direct measurement. Laboratories’ results of the QC-solutions were normalized against the target values resulting from the gravimetric preparation at BAM (QC-solution 1: 50.6 ng mL^−1^ and QC-solution 2: 180.7 ng mL^−1^). The ZEN results obtained by the participants for the unknown maize oil sample were normalized against the value assigned by BAM’s in-house study (362.3 μg kg^−1^). The three possible combinations of evaluation are displayed in Fig. S2 (ESM).

#### Evaluation criteria

Data sets within 80 to 120% for both QC-solutions and the ERM®-BC715 candidate were retained; results lying outside ±20% of the target values were excluded from further evaluation due to technical reasons. Four out of 13 laboratory data sets were excluded referring to:

- Lab B:low on both QC-solutions (66%, 61%), but high for ERM®-BC715 (148%)
- Lab F:very low on both QC-solutions (21%, 23%)
- Lab H:very low for ERM®-BC715 (12%)
- Lab I:low on QC-solution 1 (53%)

**Fig. 4:**
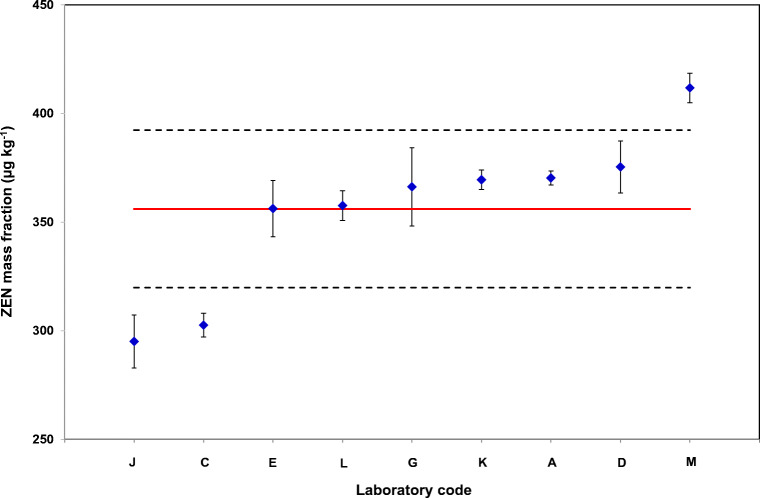
Mean values of accepted laboratory data sets of ILC for ZEN in maize germ oil (ERM®-BC715) with their corresponding standard deviations (*n* = 6) represented by error bars. The mean of all laboratory means is 356.1 μg kg^−1^ (red line), the standard deviation of the mean of means is indicated by the dotted lines

According to EC Regulation No. 401/2006 [23], the ZEN recovery of official control methods should fall within 70–120%. Eight out of 9 labs fulfilled this requirement. One lab (G) reported a ZEN recovery of 55%. However, both control solutions (100%, 102%) and the value for ERM®-BC715 (101% after correction by recovery) showed very good results, so Lab G was retained. The accepted data sets are shown in Fig. 4.

The conformity of the ILC result and the assigned value was tested using the (amended) *E*_n_ criterion on the difference between the overall laboratory mean *x*_1_ (356.1 μg kg^−1^) and the assigned value *x*_2_ (362.3 μg kg^−1^) according to Eq. 2:

2 $$ {E}_{\mathrm{n}}=\frac{\left|{x}_1-{x}_2\right|}{2\sqrt{s_{\mathrm{ILC}}^2+{u}_{\mathrm{c}}^2}} $$

*s*_ILC_: standard deviation of the mean of accepted laboratory means of ILC (36.3 μg kg^−1^)
*u*_c_: uncertainty of the assigned value from in-house study (11.0 μg kg^−1^), see Table 5

**Table 5:** Uncertainty contributions for calculation of the combined uncertainty

Uncertainty contribution		μg kg^−1^
Uncertainty of characterization	*u*_x_	4.615
Contribution from a possibly undetected inhomogeneity	*u*_bb_	8.463
Contribution from long-term stability (sufficiently stable for shelf life of 5 years, no MU contribution required)	*u*_lts_	0
Calibration uncertainty	*u*_cal_	3.268
Uncertainty of the purity of used native calibration standard^a^	*u*_pur_	2.174
Contribution from handling of samples: weighing, volumetric operations, aliquoting internal standard (pragmatic 1%)	*u*_handling_	3.623
Combined uncertainty	*u*_c_	11.0
Expanded uncertainty	*U*	22.0

^a^At the same time traceability contribution: 0.6% of certified standard concentration

The factor 2 converts the standard uncertainties in the denominator into expanded uncertainties. The resulting *E*_n_ criterion was determined to be <0.1. Because the *E*_n_ criterion is lower than the critical value (*E*_n_ = 2), the outcome of the ILC is fully consistent with the in-house certification result.

The general trend from HPLC-FLD to the more selective HPLC-MS/MS method could be observed by comparing the instrumental methods of the participants in this ILC and the previous certification study of BCR-717 [21]. While no HPLC-MS/MS method was used in BCR-717 certification study (25 out of 26 data sets based on HPLC-FLD), 4 out of 13 participants used HPLC-MS/MS in the ILC for ERM-BC715. This change is facilitated both by the availability of isotopic labelled standards that improve accuracy (trueness and precision) of HPLC-MS/MS analysis and by the ability to omit IAC clean-up traditionally used for HPLC-FLD analysis.

### Secondary characterization

It is known from previous studies that naturally occurring *trans*-ZEN in Fig. 1 (IUPAC name: (3*S*,11*E*)-14,16-Dihydroxy-3-methyl-3,4,5,6,9,10-hexahydro-1*H*-2-benzoxacyclotetradecine-1,7(8*H*)-dione) can be readily converted to its *cis*-isomer by exposure to UV-/sunlight [24]. Because this photochemical isomerization can also occur in maize oil, the candidate material was analysed for *cis*-ZEN, but this isomer could not be detected.

Moreover, the maize germ oil was also analysed for other *Fusarium* mycotoxins such as trichothecenes, modified forms of ZEN and metabolites. However, neither trichothecenes (type A: T2-/HT2- toxins; type B: DON/NIV) nor the polar modified ZEN derivatives (ZEN-sulphate, ZEN-glucoside) and the highly estrogenic metabolites α-/β-zearalenol (ZEL) could be detected or were below quantifiable concentrations. In contrast to maize powder reference materials, in which polar and nonpolar *Fusarium* mycotoxins can be found [21], maize germ oil is mainly accessible to lipophilic substances.

The mass fractions of Cd and Pb in ERM®-BC715 were found to be below 0.01 mg kg^−1^ and thus, in the case of Pb, well below the EU maximum level of 0.1 mg kg^−1^.

### Uncertainty and traceability

The combined uncertainty (*u*_c_) of ERM®-BC715 was appropriately composed from the contributions of the in-house certification study according to Eq. 3 and Table 5.

3 $$ {u}_{\mathrm{c}}^2={u}_{\mathrm{x}}^2+{u}_{\mathrm{bb}}^2+{u}_{\mathrm{lts}}^2+{u}_{\mathrm{c}\mathrm{al}}^2+{u}_{\mathrm{pur}}^2+{u}_{\mathrm{handling}}^2 $$

An expanded uncertainty (*U*) was calculated according to Eq. 4 applying a coverage factor *k* of *k* = 2, corresponding to a level of confidence of approximately 95%, as defined in GUM [25].

4 $$ U=k\cdotp {u}_{\mathrm{c}} $$

The calibration uncertainty *u*_cal_ is the uncertainty of a typical determination in the centre of a typical calibration curve. The uncertainty from handling *u*_handling_ is a combined, rather worst-case, estimate for all gravimetric and volumetric sample handling procedures. The uncertainty contribution *u*_x_ is calculated from characterization (= homogeneity) study based on Eq. 5:

5 $$ {u}_{\mathrm{x}}=\sqrt{{\left(\frac{s}{\sqrt{N}}\right)}^2+\frac{\sum \limits_i^N{s}_{\mathrm{i}}^2}{N\cdotp n}} $$

with:

*s*: standard deviation of the 20 individual mean values of the analysed ampoules
*N*: number of ampoules in homogeneity/characterization study, *N* = 20
*n*: number of replicates in homogeneity study, *n* = 5
*s*_i_: standard deviation of 5 replicates of ampoule *i*

Traceability of the certified value was directly established using HPLC-MS/MS stable isotope dilution analysis applying a certified ZEN standard (100.4 ± 0.6 μg mL^−1^, Biopure™, Tulln, Austria) as calibrant and [^13^C_18_]-isotopically labelled ZEN as internal standard. The certified mass fraction of ZEN in ERM®-BC715 is traceable via the certified calibrant used. The certified value of the calibrant is traceable to the International System of Units (SI), as stated in the respective certificate, due to the gravimetric preparation employed. Therefore, the mass fraction of ZEN in ERM®-BC715 is traceable to the SI.

## Conclusions

ERM®-BC715 represents the first CRM developed for the determination of ZEN in maize germ oil considering the EU maximum level of 400 μg kg^−1^. The production of the CRM and the characterization (homogeneity, stability) as well as the assignment of the certified property value were done at BAM in compliance with the internationally accepted procedures laid down in ISO Guide 35. The certified mass fraction and expanded uncertainty (*k* = 2) of the reference material (362 ± 22) μg kg^−1^ ZEN are traceable to the SI. The obtained mass fraction based upon the in-house study using SIDA-HPLC-ESI-MS/MS was in good agreement with the consensus value from the supporting interlaboratory comparison with 13 experienced laboratories using HPLC-MS/MS and HPLC-FLD instrumental methods. Owing to the limited number of matrix CRMs available for analysis of ZEN in similar matrices, ERM®-BC715 is intended for use in the development and validation of new analytical methods, and represents an important quality control tool for laboratories to implement and safeguard reliable measurements of ZEN in relevant food matrices.

In addition to the regulatory-limited *trans*-ZEN, future CRMs should also focus on photochemically formed *cis*-ZEN, biologically modified ZEN, e.g. sulphate and glucoside conjugates, which are naturally formed by plants, and the highly estrogenic metabolites α- (and β-) ZEL. All these derivatives contribute to the total ZEN/ZEL exposure.

## Supplementary information

ESM 1 (DOCX 60 kb)

## Data Availability

All data and material are available.
