# Supplementary material for: Development and certification of a reference material for zearalenone in maize germ oil
Source: Anal Bioanal Chem. 2021 Jul 21;413(21):5483–91. doi: 10.1007/s00216-021-03532-z (PMC8405470; doi:10.1007/s00216-021-03532-z)
Supplement: Supplementary file 1 — (DOCX 60 kb) [file 216_2021_3532_MOESM1_ESM.docx]

**Supplementary Information (SI)**

**Development and certification of a reference material
for zearalenone in maize germ oil**

Juliane Riedel, Sebastian Recknagel, Diana Sassenroth, Tatjana Mauch, Sabine Buttler, Thomas Sommerfeld, Sibylle Penk, Matthias Koch

All authors:

Bundesanstalt für Materialforschung und -prüfung (BAM), Richard-Willstätter-Straße 11, 12489 Berlin, Germany

**Fig. S1** Effective reaction rate k*_eff_* for ZEN in maize germ oil (ERM^®^-BC715) in dependence on the inverse temperature (semilogarithmic plot) by assuming an *Arrhenius* model. Solid line: linear regression over the stability data; dotted lines: upper and lower confidence limits of the regression line

**Fig. S2 (a)** Youden plot of the normalised laboratory mean values for ZEN in ERM^®^-BC715 against the normalised QC-solution 1 values. Suspected laboratories (B, F, H, I) laying outside ± 20% of the target values indicated by the green box.

**Fig. S2 (b)** Youden plot of the normalised laboratory mean values for ZEN in ERM^®^-BC715 against the normalised QC-solution 2 values. Suspected laboratories (B, F, H) laying outside ± 20% of the target values indicated by the green box.

**Fig. S2 (c)** Youden plot of the normalised QC-solution 2 values against the normalised QC-solution 1 values. Suspected laboratories (B, F, I) laying outside ± 20% of the target values indicated by the green box.
